# Supplementary material for: Snakebite Treatment in Tanzania: Identifying Gaps in Community Practices and Hospital Resources
Source: Int J Environ Res Public Health. 2022 Apr 13;19(8):4701. doi: 10.3390/ijerph19084701 (PMC9024466; doi:10.3390/ijerph19084701)
Supplement: Supplementary file 1 [file ijerph-19-04701-s001.zip › S3.pdf]

```
#Demographics (age, sex, program, location)
age<-length(a$YOB)
a$age<-(a$year - a$YOB)
age_groups <- ifelse(a$age >=20 & a$age <=25, "20-25",
                              ifelse(a$age > 25, "25+", NA))
a$age_groups<-age_groups
table(a$year, exclude=NULL)
table(a$YOB, exclude=NULL)
table(a$age)
summary(a$age)
sd(a$age, na.rm=TRUE)
table(a$sex, exclude=NULL)
table(a$program, exclude=NULL)
table(a$grewup1rural2urban, exclude=NULL)
table(a$FAOecoregions, exclude=NULL)

prop.table(table(age_groups, exclude=NULL))*100
prop.table(table(a$sex, exclude=NULL))*100
prop.table(table(a$grewup1rural2urban, exclude=NULL))*100
prop.table(table(a$FAOecoregions, exclude=NULL))*100
prop.table(table(a$program, exclude=NULL))*100

#TREATMENT
treatment.string<-paste(b$treatment, collapse=",")
treatment.vector<-strsplit(treatment.string, ",")[[1]]
treatment.vector.clean<-gsub(" ", "", treatment.vector)
table(treatment.vector.clean)
sort(table(treatment.vector.clean))
sort(table(treatment.vector.clean))/312*100

#Classify treatment as traditional vs allpathic
traditional <-c("tie", "washwound", "washwound.soap", "washwound.water",
"cutbittenarea", "suckoutvenom", "amputation", "snakestone", "snakestone.charcoal",
"snakestone.milk", "coin", "coin.100shilllingcoin", "coin.50shillingcoin", "coin.silver",
"coin.gold", "traditionalmedicine", "traditionalmedicine.msongwa",
"traditionalmedicine.milk", "traditionalmedicine.kerosine", "traditionalmedicine.herb",
"traditionalmedicine.amura", "traditionalmedicine.freshdogear",
"traditionalmedicine.eggyolk", "traditionalmedicine.ashes", "hometreatment",
"prayers", "nohospitalavailable")
allopathic <-c("miscconv.", "miscconv.tiedonotblock", "miscconv.notie",
"miscconv.maintainhygiene", "miscconv.avoidmovement", "bandage", "nobandage",
"noalcohol", "miscconv.injuredpartbelowheartlevel", "miscconv.staycalm", "medicine",
"hospital", "hospital.iv", "hospital.ringerslactate", "hospital.antiallergy",
"hospital.antiallergy.hydrocortisone", "hospital.antitetanus", "hospital.vaccination",
"hospital.antirabies", "pav", "identifysnake", "identifydanger", "firstaid",
"miscconv.guidelines", "removeteeth")
b$treatment <- as.character(b$treatment)
b$tradallop <- sapply(strsplit(b$treatment, ',\\s*'), function(x) {x <- na.omit(x)
if(all(x %in% traditional)) 'traditional'
else if(all(x %in% conventional)) 'allopathic'
```

```
else 'both'
})
b$tradallop
prop.table(table(b$tradallop))*100 #NAs not minused
table(b$tradallop)
z<-c(144, 22, 131, 15)/312 *100
z

#Hospital facilities available (IV, AR, MOH, PAV, WBC)
table(a$IV, exclude=NULL)
table(a$AR, exclude=NULL)
table(a$MOH, exclude=NULL)
table(a$PAV, exclude=NULL)
table(a$WBC, exclude=NULL)

prop.table(table(a$IV, exclude=NULL))*100
prop.table(table(a$AR, exclude=NULL))*100
prop.table(table(a$MOH, exclude=NULL))*100
prop.table(table(a$PAV, exclude=NULL))*100
prop.table(table(a$WBC, exclude=NULL))*100

prop.table(table(a$IV))*100
prop.table(table(a$AR))*100
prop.table(table(a$MOH))*100
prop.table(table(a$PAV))*100
prop.table(table(a$WBC))*100

#How many hours before the patient ar-rived at hospital for treatment?
table(c$hrstohospital, exclude=NULL)
(table(c$hrstohospital, exclude=NULL))*100/69
summary(c$hrstohospital)
sd(c$hrstohospital, na.rm=TRUE)

#Which of the following was done? (Before arrival to the hospital)
table(c$X41athospital)
X41athospital<-paste(c$X41athospital, collapse=",")
X41athospital.string<-paste(c$X41athospital, collapse=",")
X41athospital.vector<-strsplit(X41athospital.string, ",")[[1]]
X41athospital.vector.clean<-gsub(" ", "", X41athospital.vector)
table(X41athospital.vector.clean, exclude=NULL)
sort(table(X41athospital.vector.clean, exclude=NULL))
(sort(table(X41athospital.vector.clean, exclude=NULL)))/69*100

#Did the patient die?
table(c$death, exclude=NULL)
(table(c$death, exclude=NULL))/69*100

#Was the patient left with lasting damage?
table(c$lastingdamage, exclude=NULL)
(table(c$lastingdamage, exclude=NULL))/69*100
```

```
##Explain.
table(c$explaindamage)
explaindamage<-paste(c$explaindamage, collapse=",")
explaindamage.string<-paste(c$explaindamage, collapse=",")
explaindamage.vector<-strsplit(explaindamage.string, ",")[[1]]
explaindamage.vector.clean<-gsub(" ", "", explaindamage.vector)
table(explaindamage.vector.clean, exclude=NULL)
sort(table(explaindamage.vector.clean, exclude=NULL))
(sort(table(explaindamage.vector.clean, exclude=NULL)))/69*100

#confint
prop.test(x=15, n=312, conf.level=0.95, correct = FALSE)
library (Hmisc)
binconf(x=15, n=312, alpha=0.05)

#Table 1 Demographics
binconf(x=c(239, 67), n=306, alpha=0.05) #age
binconf(x=c(185, 124),n=309, alpha=0.05) #gender
binconf(x=c(242, 67),n=309, alpha=0.05) #urban, rural
binconf(x=c(29, 47, 70, 44, 101),n=291, alpha=0.05) #location
binconf(x=c(245, 63), n=308, alpha=0.05) #program

#Table 2 First aid methods
binconf(x=c(131, 22, 144), n=297, alpha=0.05)

#Table 3. Availability of hospital facilities.
binconf(x=c(24, 8), n=24+8, alpha=0.05) #IV
binconf(x=c(17, 14), n=31, alpha=0.05) #AR
binconf(x=c(13, 18), n=31, alpha=0.05) #MOH, PAV
binconf(x=c(15, 16), n=31, alpha=0.05) #WBC

#Table 4
binconf(x=c(11, 12, 6, 6, 6), n=41, alpha=0.05) #Number of hours
binconf(x=c(4, 20, 43, 18, 3, 15, 38, 13, 4, 9), n=64, alpha=0.05) #What was done
before arrival at hospital
binconf(x=c(8, 50), n=58, alpha=0.05) #Death
binconf(x=c(13, 28), n=41, alpha=0.05) #lasting damage
binconf(x=18, n=41, alpha=0.05) #3 or more hours after a bite

#Barplot traditional
b<-read.csv("~/Dropbox/Notes, Data/4 Traditional graph.csv", header=TRUE,
sep=";", dec=".")
str(b)
b$Percentageofpeople <- (b$Numberofpeople)/297 *100 #add percentage of people
b$ci_lo <- c(41, 35, 29, 11, 5, 3, 3) #add 95% conf int low
b$ci_hi <- c(52, 46, 40, 19, 11, 7, 7) #add 95% conf int high
str(b)
p<-ggplot(b, aes(x=reorder(Traditional, Percentageofpeople), y=
Percentageofpeople)) + geom_bar(stat="identity", fill="pink2",
```

```
width=0.5)+labs(y="Percentage of people (N = 297)") + labs (x="Traditional
practices") +
   geom_errorbar(aes(x=Traditional, ymin=ci_lo, ymax=ci_hi), width=0.2,
colour="black", alpha=0.5, size=1) +
   scale_y_continuous(breaks = scales::pretty_breaks(n = 5))+
   theme(axis.text=element_text(size = 17), axis.title=element_text(size = 20),
axis.title.y=element_text(vjust=6), plot.margin = margin(20, 20, 20, 30))
p+coord_flip()

#Barplot allopathic
a<-read.csv("~/Dropbox/Notes, Data/4 Allopathic graph.csv", header=TRUE, sep=";",
dec=".")
str(a)
a$Percentageofpeople <- (a$Numberofpeople)/297 *100 #add percentage of people
a$ci_lo <- c(45, 10, 3, 3, 1, 1, 1) #add 95% conf int low
a$ci_hi <- c(56, 18, 9, 8, 4, 4, 5) #add 95% conf int high
str(a)
p<-ggplot(a, aes(x= reorder(Allopathic, Percentageofpeople), y=
Percentageofpeople)) + geom_bar(stat="identity", fill=rgb(0.1,0.4,0.5,0.7),
width=0.5)+labs(y="Percentage of people (N = 297)") + labs (x="Allopathic practices")
+
   geom_errorbar( aes(x=Allopathic, ymin=ci_lo, ymax=ci_hi), width=0.2,
colour="black", alpha=0.5, size=1) + scale_y_continuous(breaks =
scales::pretty_breaks(n = 5)) +
   theme(axis.text=element_text(size = 17), axis.title=element_text(size = 20),
axis.title.y=element_text(vjust=6), plot.margin = margin(20, 20, 20, 30))
p+coord_flip()
```
